# Crosstalk between HIV and hepatitis C virus during co-infection

**DOI:** 10.1186/1741-7015-10-32

**Published:** 2012-04-03

**Authors:** Paul J Rider, Fenyong Liu

**Affiliations:** 1 School of Public Health, University of California, Berkeley, CA 94720, USA; 2 Program in Comparative Biochemistry, University of California, Berkeley, CA 94720, USA

**Keywords:** Co-infection, HIV, HCV, IP-10

## Abstract

An estimated one-third of individuals positive for HIV are also infected with hepatitis C virus (HCV). Chronic infection with HCV can lead to serious liver disease including cirrhosis and hepatocellular carcinoma. Liver-related disease is among the leading causes of death in patients with HIV, and individuals with HIV and HCV co-infection are found to progress more rapidly to serious liver disease than mono-infected individuals. The mechanism by which HIV affects HCV infection in the absence of immunosuppression by HIV is currently unknown. In a recent article published in *BMC Immunology*, Qu *et al. *demonstrated that HIV tat is capable of inducing IP-10 expression. Further, they were able to show that HIV tat, when added to cells, was able to enhance the replication of HCV. Importantly, the increase in HCV replication by tat was found to be dependent on IP-10. This work has important implications for understanding the effect HIV has on the outcome of HCV infection in co-infected individuals. The findings of Qu *et al. *may inform the design of intervention and treatment strategies for co-infected individuals.

Please see related article: http://www.biomedcentral.com/1471-2172/13/15.

## Background

A significant number of individuals with HIV are co-infected with serious human pathogens such as hepatitis C virus (HCV), which infects up to 170 million people globally [1]. Due to similar routes of infection for HIV and HCV, it is estimated that up to a third of people with HIV are co-infected with HCV [1-3]. With an estimated 34 million cases of HIV infection worldwide [4], this represents a disease burden for individuals with co-infection of HIV and HCV of greater than 10 million people. Prior to the era of effective anti-retroviral therapy, patients with HIV did not live long enough to suffer disease caused by chronic infection with HCV. However, as a result of the effectiveness of current anti-retroviral therapies, these individuals with HIV and HCV co-infection are now living long enough to be affected by disease caused by chronic HCV infection.

Liver-related disease is a leading cause of death in individuals infected with HIV, mostly due to co-infection with HCV [1,5,6]. There is an abundance of evidence that the normal progression of HCV pathogenesis is altered in the presence of HIV [7-10]. These studies have shown that individuals co-infected with HIV and HCV possess higher levels of HCV RNA, and progress more rapidly to serious liver disease, including cirrhosis and hepatocellular carcinoma [11]. Individuals with an HIV and HCV co-infection are also more resistant to standard HCV treatments than patients with an HCV mono-infection. For example, successful treatment with interferon/ribavirin in patients with an HCV genotype 1 mono-infection reaches 50% to 80% while in co-infected individuals effective treatment is reduced to 20% to 35% [1]. The mechanisms by which HIV affects HCV infection in individuals with a co-infection are currently unknown.

### IP-10 and HIV and HCV co-infection

A number of research groups have sought to determine how HIV infection affects HCV in individuals that are co-infected. Multiple studies have identified elevated IP-10 (CXCL10) levels as a negative prognostic indicator for both HCV infection and HIV and HCV co-infection [12-14]. Further, elevated IP-10 levels were associated with the poor response to interferon/ribavirin therapy noted above [15]. IP-10 is a CXC chemokine that binds to the CXCR3 immune system receptor present on a number of cells, including monocytes, natural killer cells and T-cells [16]. Binding of IP-10 to CXCR3 induces chemotaxis of stimulated cells to sites of infection or injury. It is thought that elevated IP-10 could recruit T-cells from the periphery and within the liver, mediating the damage seen in cirrhosis and hepatocellular carcinoma [17]. The mechanism by which elevated levels of IP-10 may affect HCV infection in individuals with HIV and HCV co-infection is not known.

Work by Qu *et al.*, published in *BMC Immunology*, describes a potential molecular mechanism for the influence HIV has on HCV infection in individuals with HIV and HCV co-infection. Specifically, Qu *et al. *present findings describing a potential cause-and-effect relationship between HIV infection and increased HCV replication. HIV tat is shown to increase levels of IP-10 mRNA and protein and consequently enhance HCV replication. Importantly, IP-10 induction by tat is shown to be necessary and sufficient to increase HCV replication levels. Their data further demonstrate a synergism in the ability of HIV and HCV to induce IP-10: hepatocytes that were both infected with HCV and treated with HIV tat induced higher levels of IP-10 mRNA and protein than either HCV infection or treatment with HIV tat alone.

### Understanding co-infection

It is challenging for many to imagine a situation in which both HIV and HCV would find themselves in the same cell and thus in a position to affect the replication of one another. However, specialized immune cells, called peripheral blood mononuclear cells and Kupffer cells, are sites of infection for both HIV and HCV and the possibility exists that both HIV and HCV could find themselves in the same cell in an individual with a co-infection [18]. Experiments should be carried out to determine if tat-induced IP-10 can affect HCV replication in Kupffer cells and peripheral blood mononuclear cells. However, an interesting implication of the work presented by Qu *et al. *is that HIV does not need to be present in the same cell to exert an effect on HCV infection. Both tat [19] and IP-10 can act on cells distant from the site of HIV infection.

The work by Qu *et al. *poses many intriguing and important questions. Certainly it is important to determine the mechanisms by which HIV upregulates IP-10 expression and, more importantly, how IP-10 enhances HCV replication. CXCR3 receptor expression is not known to be expressed on hepatocytes [16]; thus, in the experiments described by Qu *et al.*, IP-10 may be acting through an as yet unknown mechanism. Additionally, it was recently shown that induced IP-10 in patients with HCV infection was expressed in a truncated, antagonist form [20]. Cellular proteinases responsible for the conversion of IP-10 to its antagonist form are presumably absent in the experiments performed by Qu *et al. *It will be interesting to determine which form is important in individuals who are co-infected and whether the antagonistic IP-10 has a similar influence on HCV replication as found in the paper.

There are reports of a genotype-specific response to HCV treatments [15] and it will be necessary to determine whether IP-10's effect on HCV is genotype specific. Similar experiments will need to be performed using multiple genotypes. Further, these findings will need to be addressed in an *in vivo *setting, such as a humanized mouse or primate model, to assess the relevance of these findings to the pathogenesis of HIV and HCV co-infection. IP-10 is the only chemokine examined by Qu *et al.*; however, multiple chemokines are found to be differentially regulated in individuals with HCV-associated liver disease [17]. It will be important to determine if other chemokines are able to enhance HCV replication as shown in the paper by Qu *et al.*

An important consideration for HIV co-infection studies is whether the effect measured is due to HIV or whether the effect can be attributed to the immunodeficiency that defines AIDS [1]. The relationship between HIV and pathogens such as HCV in individuals with a co-infection is not simply one of opportunism, whereby the immunodeficiency caused by HIV creates a situation in which normally non-pathogenic organisms can cause disease. Rather, the direct or indirect interplay between pathogens in individuals with co-infection is presumed to alter the normal progression of the disease seen in individuals with a mono-infection. In the study by Qu *et al.*, individuals who had progressed to AIDS (as determined by CD4 and CD8 counts) were included in the cohort to show a correlation between HIV, HCV and IP-10 levels. It will be important in future experiments to exclude individuals with an immunodeficiency as this confounds the interpretation of the effect of HIV infection on HCV viral load and IP-10 levels in those individuals. The low CD4 counts are likely responsible for some increase in HCV replication and, perhaps, the IP-10 levels reported. Still, the finding that upregulation of IP-10 expression by HIV tat can enhance the replication of HCV illuminates IP-10 and HIV tat as targets for therapeutic intervention in individuals co-infected with HIV and HCV.

## Conclusions

The work presented by Qu *et al. *advances our understanding of the relationship between HIV and HCV in individuals with a co-infection, an area that is extremely important yet poorly understood. These findings have important implications for the rational design of therapeutic intervention to treat or prevent HCV liver disease in both individuals with HIV and HCV co-infection as well as those with an HCV mono-infection.

## Competing interests

The authors declare that they have no competing interests.

## Authors' contributions

PJR and FL wrote, read and approved the final manuscript.

## Authors' information

PJR and FL are currently affiliated with and conducting research at School of Public Health, University of California at Berkeley, USA.

## Pre-publication history

The pre-publication history for this paper can be accessed here:

http://www.biomedcentral.com/1741-7015/10/32/prepub
